# Response of male adult *Aedes* mosquitoes to gamma radiation in different nitrogen environments

**DOI:** 10.3389/fbioe.2022.942654

**Published:** 2022-09-12

**Authors:** Nanwintoum Séverin Bimbilé Somda, Hanano Yamada, Carina Kraupa, Wadaka Mamai, Hamidou Maiga, Simran Singh Kotla, Thomas Wallner, Claudia Martina, Jeremy Bouyer

**Affiliations:** ^1^ Insect Pest Control Laboratory, Joint FAO/IAEA Centre of Nuclear Techniques in Food and Agriculture, International Atomic Energy Agency, Vienna, Austria; ^2^ Unité de Formation et de Recherche en Sciences et Technologies (UFR/ST), Université Norbert ZONGO (UNZ), Koudougou, Burkina Faso; ^3^ Institut de Recherche en Sciences de la Santé/Direction Régionale de l’Ouest (IRSS/DRO), Bobo-Dioulasso, Burkina Faso; ^4^ Institut de Recherche Agricole pour le Développement (IRAD), Yaoundé-Messa, Cameroun

**Keywords:** sterile insect technique, irradiation, *Aedes aegypti*, *Aedes albopictus*, anoxia

## Abstract

The developmental stage of the mosquito is one of the main factors that affect its response to ionizing radiation. Irradiation of adults has been reported to have beneficial effects. However, the main challenge is to immobilize and compact a large number of adult male mosquitoes for homogenous irradiation with minimal deleterious effects on their quality. The present study investigates the use of nitrogen in the irradiation of adult *Aedes albopictus* and *Ae. aegypti*. Irradiation in nitrogen (N_2_) and in air after being treated with nitrogen (PreN_2_) were compared with irradiation in air at gamma radiation doses of 0, 55, 70, 90, 110, and 125 Gy. In both species, approximately 0% egg hatch rate was observed following doses above 55 Gy in air versus 70 Gy in PreN_2_ and 90 Gy in N_2_. Males irradiated at a high mosquito density showed similar egg hatch rates as those irradiated at a low density. Nitrogen treatments showed beneficial effects on the longevity of irradiated males for a given dose, revealing the radioprotective effect of anoxia. However, irradiation in N_2_ or PreN_2_ slightly reduced the male flight ability. Nitrogen treatment was found to be a reliable method for adult mosquito immobilization. Overall, our results demonstrated that nitrogen may be useful in adult *Aedes* mass irradiation. The best option seems to be PreN_2_ since it reduces the immobilization duration and requires a lower dose than that required in the N_2_ environment to achieve full sterility but with similar effects on male quality. However, further studies are necessary to develop standardized procedures including containers, time and pressure for flushing with nitrogen, immobilization duration considering mosquito species, age, and density.

## 1 Introduction

The sterile insect technique (SIT), a species-specific and environment-friendly method, is a promising technique for the area-wide integrated management of vector mosquitoes, which cause diseases such as dengue, yellow fever, and Zika (Dyck et al., 2021). Indeed, many field trials to demonstrate the effectiveness of the SIT against *Aedes aegypti* and *Aedes albopictus* have shown satisfactory results on a small scale (Bellini et al., 2013, 2021; Gato et al., 2021; Becker et al., 2022). Although significant advances have been made in its development (Culbert et al., 2018; Bimbilé Somda et al., 2019; Mamai et al., 2019; Mamai et al., 2020; Yamada et al., 2019; Bouyer et al., 2020; Maïga et al., 2020), there is considerable scope for further development, especially for large-scale implementation. The SIT package includes mosquito mass rearing, sex separation, male sterilization, transport, and release (Dyck et al., 2021). Sterilizing large numbers of males in a reliable manner while maintaining their quality remains one of the most challenging steps. Chemosterilization (Patterson et al., 1970), ionizing radiation (Patterson et al., 1975), and genetic manipulations (Catteruccia et al., 2009) are the main methods explored for mosquito sterilization. However, because of limited effectiveness or ethical, health, and environmental concerns, ionizing radiation, which does not release residues that could be harmful to human health or the environment (Helinski et al., 2009), is the most used method in current SIT programs. Gamma radiation from a ^60^Co or ^137^Cs source and X-ray radiation are commonly used because of their high energy and penetration (Helinski et al., 2009). However, the overall response of mosquitoes to radiation, as with all biological material, has been shown to be affected by many critical factors, including the radiation source, dose rate, dose amount, environment during irradiation, mosquito species, strain, life stage, and handling procedures (Helinski et al., 2009; Yamada et al., 2019; Yamada et al., 2020). For example, irradiation of eggs or larvae causes a high mortality rate even at low irradiation doses and cannot be considered for the SIT programs (Wakid et al., 1976). The pupal and adult stages were found to be more eligible for irradiation (Helinski et al., 2006; Helinski et al., 2009). However, optimal pupae irradiation has many requirements: synchronized pupal production is needed for collection in a small age range, and generally, older pupae (>36 h) should be used to avoid variability in induced sterility. Indeed, younger pupae are more radiosensitive than older pupae, and irradiating young pupae also results in increased somatic damage and mortality (Yamada et al., 2019). Larger numbers of pupae also need to either be submerged in water to allow compacting the pupae in a small container without crushing those at the bottom (note that the pupae would be subjected to hypoxia) or undergo relaxed compaction (single layers of pupae) to avoid crushing, but where pockets of hypoxia can form in which a subset of the pupae may be underdosed (Yamada et al., 2020). These conditions are not easy to achieve or reproduce, especially for mass irradiation. While efforts are still being devoted to overcome these difficulties, the focus is now shifting to irradiation during the adult stage. It has been reported that adult *Aedes* mosquitoes are similarly or slightly more radiosensitive than old pupae with a better quality after irradiation in some cases (Du et al., 2019; Ernawan et al., 2022). However, the main challenge is the immobilization and compaction of a large number of adult males for homogenous irradiation with minimal deleterious effects on their downstream quality. In general, cold temperatures are used to knock down the mosquitoes (Zhang et al., 2020; Ernawan et al., 2022). However, the quality of the sterile males can be compromised if the chilling temperatures and duration are not carefully controlled. Indeed, many studies on the effects of chilling on insect quality have reported negative effects (reviewed in Yamada et al., 2022). Aiming for a more reliable outcome regarding quality following adult mosquito irradiation, the role of nitrogen has been investigated (Helinski et al., 2009). Beneficial effects of the use of nitrogen in insect irradiation have been reported in fruit flies (Fisher, 1996) and tsetse flies (Vreysen, 1995; Mutika and Parker, 2006). However, its impact on mosquitoes is debated. Indeed, tests performed on *Anopheles gambiae* pupae and *Culex quinquefasciatus* pupae and adults showed no beneficial effect (Curtis, 1976; El-Gazzar et al., 1983)*.* In contrast, the irradiation of adult male *Ae. aegypti* in nitrogen resulted in better competitiveness compared with irradiation in air, although higher doses were required to achieve full sterility (Hallinan and Rai, 1973). A recent study (Yamada et al., 2022) involving *Ae. albopictus* also showed a radioprotective effect of nitrogen at 45 Gy.

The present study aims to further investigate the possibility to use nitrogen in the mass irradiation of adult *Ae. albopictus* and *Ae. aegypti* mosquitoes in order to optimize the effectiveness of the SIT. Two nitrogen treatments were assessed in comparison to irradiation in air. The irradiation dose–response curves under these nitrogen treatments were determined considering gamma radiation doses of 0–125 Gy at low and high mosquito densities. The effects on male flight ability and longevity were further evaluated. A preliminary study was carried out to determine knock-down and wake-up times of adult male *Aedes* mosquitoes following their exposure to nitrogen.

## 2 Materials and methods

### 2.1 Mosquito strains

Experiments were carried out on *Ae. aegypti* and *Ae. albopictus* species*.* The strains used were maintained at the Insect Pest Control Laboratory (IPCL) of the joint FAO/IAEA Centre of Nuclear Techniques in Food and Agriculture, Seibersdorf, Austria, under rearing protocols developed at the IPCL (FAO/IAEA, 2020b).

### 2.2 Determination of the response of adult male *Aedes albopictus* following exposure to nitrogen

A preliminary study was carried out to determine the time to the first stand-up and the first flight of adult male *Aedes* mosquitoes following increasing durations of immobilization with di-nitrogen (N_2_) gas. Eight immobilization durations were tested: 1, 5, 10, 20, 30, 40, 50, and 60 min. The mosquitoes were placed in N_2_ in gas-tight headspace vials (20 ml) with screw tops with PTFE/silicone septa (Merck KGaA, Darmstadt, Germany). Five adult *Ae. albopictus* males, aged 1–2 days old, were transferred to each vial. After the mosquitoes were transferred, the vials were closed with the screw tops, and the tops were sealed with PTFE Thread Seal Tape (Sigma-Aldrich, USA) before flushing with N_2_, as described by Yamada et al. (2022). To flush N_2_ in the vial, two syringe needles were inserted from the top. One served to add the N_2_ and the other served as an outlet for the air. Following exposure to N_2_, the mosquitoes from each vial were released into an individual BugDorm cage (15 cm^3^ × 15 cm^3^ × 15 cm^3^) (MegaView Science Co. Ltd., Taichung 40762, Taiwan). The times between the release and the first mosquito to stand up as well as the first mosquito to fly were recorded. Three biological replicates with three technical replicates were performed for all exposure durations.

### 2.3 Irradiation source and dosimetry

Irradiation was performed in a ^60^Co gamma irradiator, Gammacell 220 (Nordion Ltd., Kanata, Ontario, Canada), which had a dose rate of 65 Gy/min during the experiments. Gafchromic HD-V2 film (Ashland Advanced Materials, Bridgewater NJ, United States) was used to verify the dose received by the mosquitoes (IAEA, 2004). Three pieces of HD film were individually packed in small (2 cm × 2 cm) paper envelopes and placed in the same position as the mosquito samples in the irradiation container. The films were read using an optical density reader (DoseReader 4, RadGen, H-1118 Budapest, Sasadi út 36, Hungary) after 24 h of development.

### 2.4 Irradiation doses and environments

Mosquitoes were exposed to six irradiation doses, 0, 55, 70, 90, 110, and 125 Gy, in three environments, air, nitrogen, and pretreated with nitrogen. For irradiation in air, the mosquitoes were kept in vials in the ambient atmosphere. For irradiation in nitrogen (N_2_), the air in the vial was replaced with N_2_ gas. The immobilization duration in the nitrogen was 15–20 min, including the irradiation exposure duration. The pretreated with nitrogen (Pre.Nitrogen or PreN_2_) treatment involved immobilizing the mosquitoes with nitrogen for 10–15 min and then replacing the nitrogen with air by keeping the vial open for 20–30 s prior to irradiation.

Two- to three-day-old virgin male *Aedes* were knocked down in a cold room (6°C) for 10–15 min and then transferred into gas-tight headspace vials (20 ml) with screw tops with PTFE/silicone septa (Merck KGaA, Darmstadt, Germany) for irradiation. These knockdown conditions are known to have no impact on male quality (Culbert et al., 2019). For irradiation in air, the vials were only covered with a piece of mosquito bed-net instead of the normal screw top. For both types of nitrogen treatments, the vials containing the mosquitoes were closed with screw tops and the tops were sealed with PTFE Thread Seal Tape (Sigma-Aldrich, USA) before filling with N_2_. N_2_ was flushed in the vial as described above.

### 2.5 Determination of the dose–response curves following irradiation of low-density adult *Aedes* under different nitrogen environments

The dose–response curves were determined for both *Aedes* species at low-density irradiation considering the six doses and three environments. Each vial contained 20 males. After irradiation, the mosquitoes from each vial were immediately transferred to a BugDorm cage (15 cm^3^ × 15 cm^3^ × 15 cm^3^) (MegaView Science Co. Ltd., Taichung 40762, Taiwan) containing 20 virgin females from the same male collection cohort. Two consecutive blood meals were offered to the females on days 3 and 4 following the irradiation day. Oviposition cups were placed inside the cages on day 5 and were collected on day 10. Eggs were allowed to mature and were stored following the IAEA guidelines (FAO/IAEA, 2020a). On day 10 following egg collection, eggs were allowed to hatch for over 48 h in boiled and cooled (deoxygenated) water with a pinch of larval food (for *Ae. aegypti*) or in a hatching solution containing nutrient broth and brewer’s yeast (for *Ae. albopictus*) (FAO/IAEA, 2020a). To determine their hatching status, eggs were checked under a binocular microscope and counted as either hatched or nonhatched. The egg hatch rate was determined as the percentage of hatched eggs based on the total number of eggs checked. Two biological replicates with two technical replicates each were performed for each treatment and each *Aedes* species.

### 2.6 Determination of the effects of irradiation of adult male *Aedes* at high density in different nitrogen environments on egg hatch rate and male longevity

Both *Aedes* species were irradiated at a high mosquito density considering the six radiation doses and three environments. The number of males in the vial was increased to 1000–1200.

#### 2.6.1 Determination of the effect on egg hatch rate

From the batches of mosquitoes irradiated at a high density, 50 males were randomly sampled from each vial and were transferred to a BugDorm cage (15 cm^3^ × 15 cm^3^ × 15 cm^3^) containing 50 virgin females from the same cohort of pupal collection. Three replicates were performed for all the treatments. Two consecutive blood meals were offered to the females on days 3 and 4 following the day the males were added to the cages. Egg collection, storage, and hatching were performed as described in the experiment above. However, to determine the egg hatch rate, the hatching status of 100 eggs randomly selected from each replicate was checked under a binocular microscope.

#### 2.6.2 Determination of the effect on male survival

Longevity was measured for males that were allowed to mate with females (50 males + 50 females) for the hatch rate determinations. Male mortality was recorded daily, except on weekends, from the day the male mosquitoes were added to the cages with females (irradiation day) until day 21 postemergence. Three replicates were performed for each treatment and each mosquito species.

#### 2.6.3 Determination of the effect on male flight ability

After the high-density irradiation, males from each vial were transferred to a 30 cm^3^ × 30 cm^3^ × 30 cm^3^ BugDorm cage and were allowed to recover for 1 day prior to the flight test. A sugar solution (10%) was provided. Flight ability tests were performed following the methods developed by Culbert et al. (2018). Three replicates were performed for each treatment and each species. For each replicate, approximately 100 males were randomly sampled and introduced into the flight test device. After 2 h, the numbers of escapees and nonescapees were recorded. The flight ability for each replicate was determined as the percentage of escapees based on the total number of males introduced into the flight test device.

### 2.7 Statistical analysis

Statistical analyses were performed using R software (version 4.1.2) (Chambers, 2008). Data were analyzed by mosquito species. The irradiation environment and dose (considering dose, log_10_ (dose), or log_10_ (dose +1)) were considered as explanatory variables. The egg hatch rate and flight ability were analyzed using binomial generalized linear mixed models fit by maximum likelihood (Laplace approximation) (Bolker et al., 2009), with the proportion of hatched eggs and the proportion of escaped males as response variables and the replicates as random effects. Male longevity was analyzed using mixed-effects Cox regression models with irradiation dose and the environment as explanatory variables. The best models were selected based on the lowest value of Akaike information criterion (Hurvich and Tsai, 1995; Burnham and Anderson, 1998). The response variables, time to the first stand-up and first flight, were analyzed using linear mixed-effect models with immobilization duration as the explanatory variable and the replicate as random effects.

## 3 Results

### 3.1 Response of adult male *Aedes albopictus* following exposure to nitrogen

**FIGURE 1:**
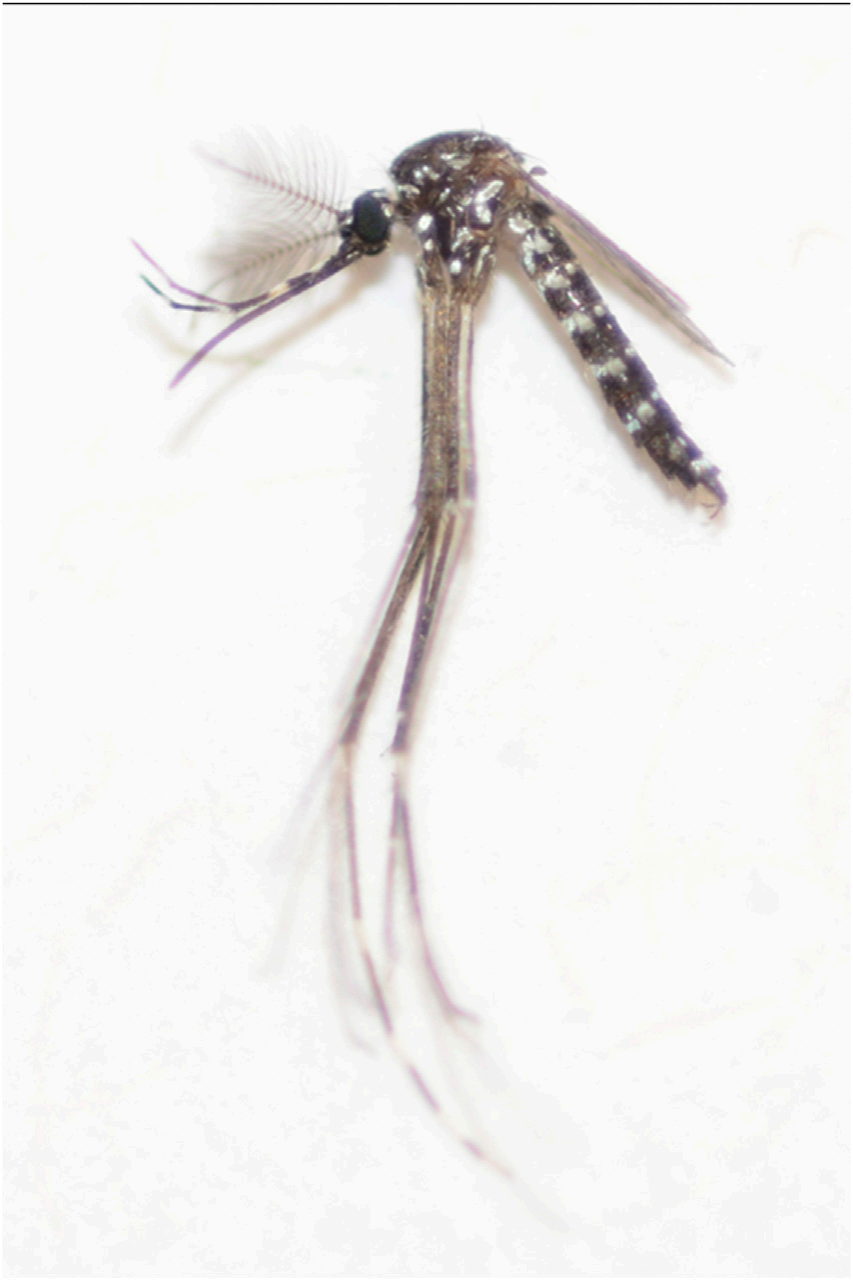
Male *Aedes albopictus* mosquito after immobilization with nitrogen.

The knockdown of adult males occurred almost immediately after the replacement of air by N_2_ in the vial. The males were completely immobilized in the N_2_ atmosphere, and their legs were stretched out straight, as shown in Figure 1.

**FIGURE 2:**
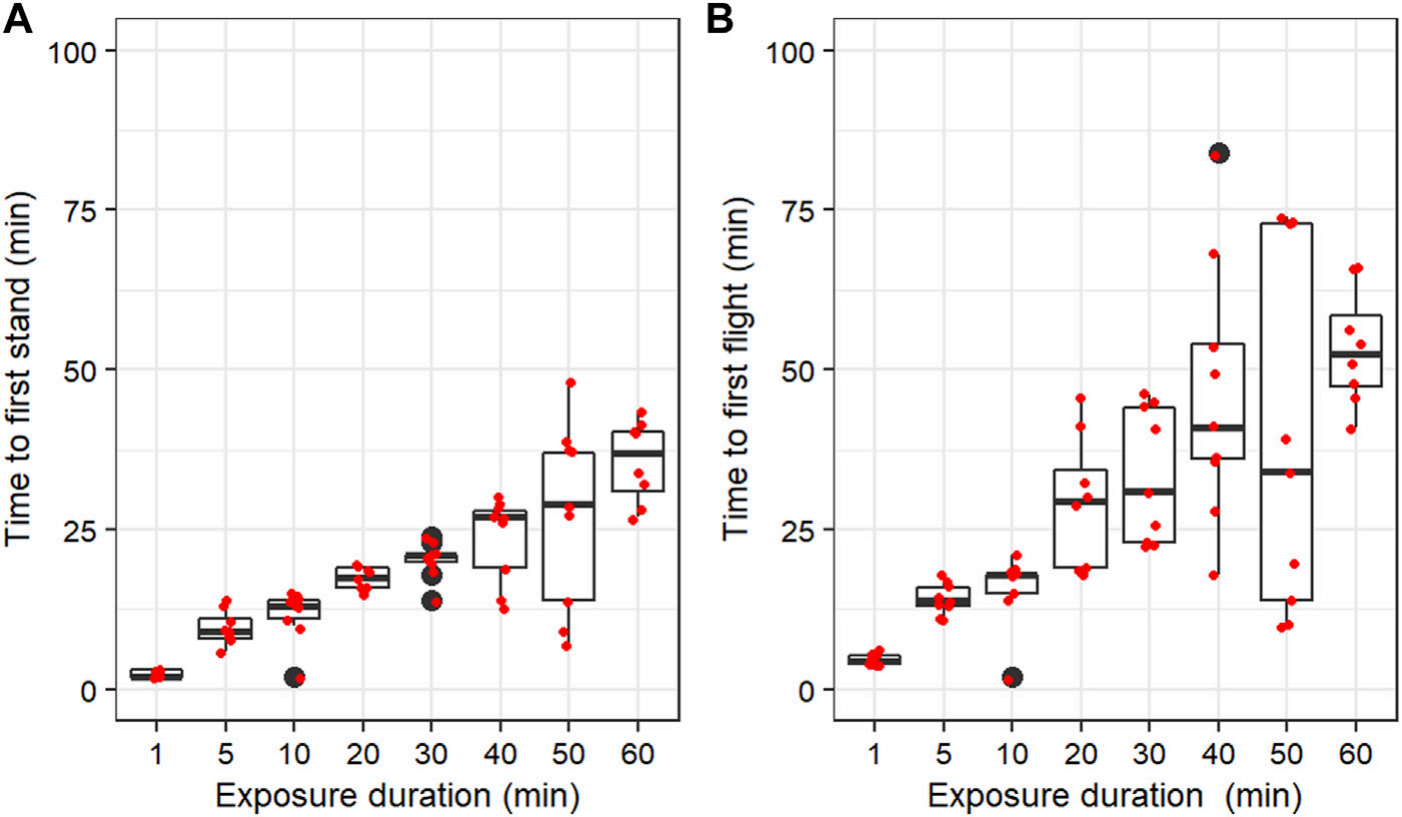
Time to first stand-up **(A)** and first flight **(B)** following exposure to nitrogen. Boxplots present the median values and quartiles, horizontal bars the 95% percentiles, black dots the minimal and maximal values, and red dots the replicate values.

The time for the male mosquitoes to stand up (Figure 2A) or fly (Figure 2B) ranged between 1 and 84 min and significantly increased with the immobilization duration (Table 1). In addition, larger variances among replicates were observed with longer immobilization durations (Figure 2).

**TABLE 1:** Fixed-effects coefficients of linear mixed-effect models of the impact of immobilization duration of male mosquitoes with nitrogen on the time to first stand-up and first flight in *Aedes albopictus*.

		Value	Std. Error	DF	t-value	*p*-value
Time to first stand-up	(Intercept)	5.800643	1.9458961	59	2.980962	0.0042
	Duration	0.469771	0.0366387	59	12.821723	< 0.0001
Time to first flight	(Intercept)	9.594257	3.967283	59	2.418344	0.0187
	Duration	0.731252	0.08174	59	8.946059	< 0.0001

### 3.2 Dose–response curves of *Aedes* following irradiation of low-density adult males in different nitrogen environments

**FIGURE 3:**
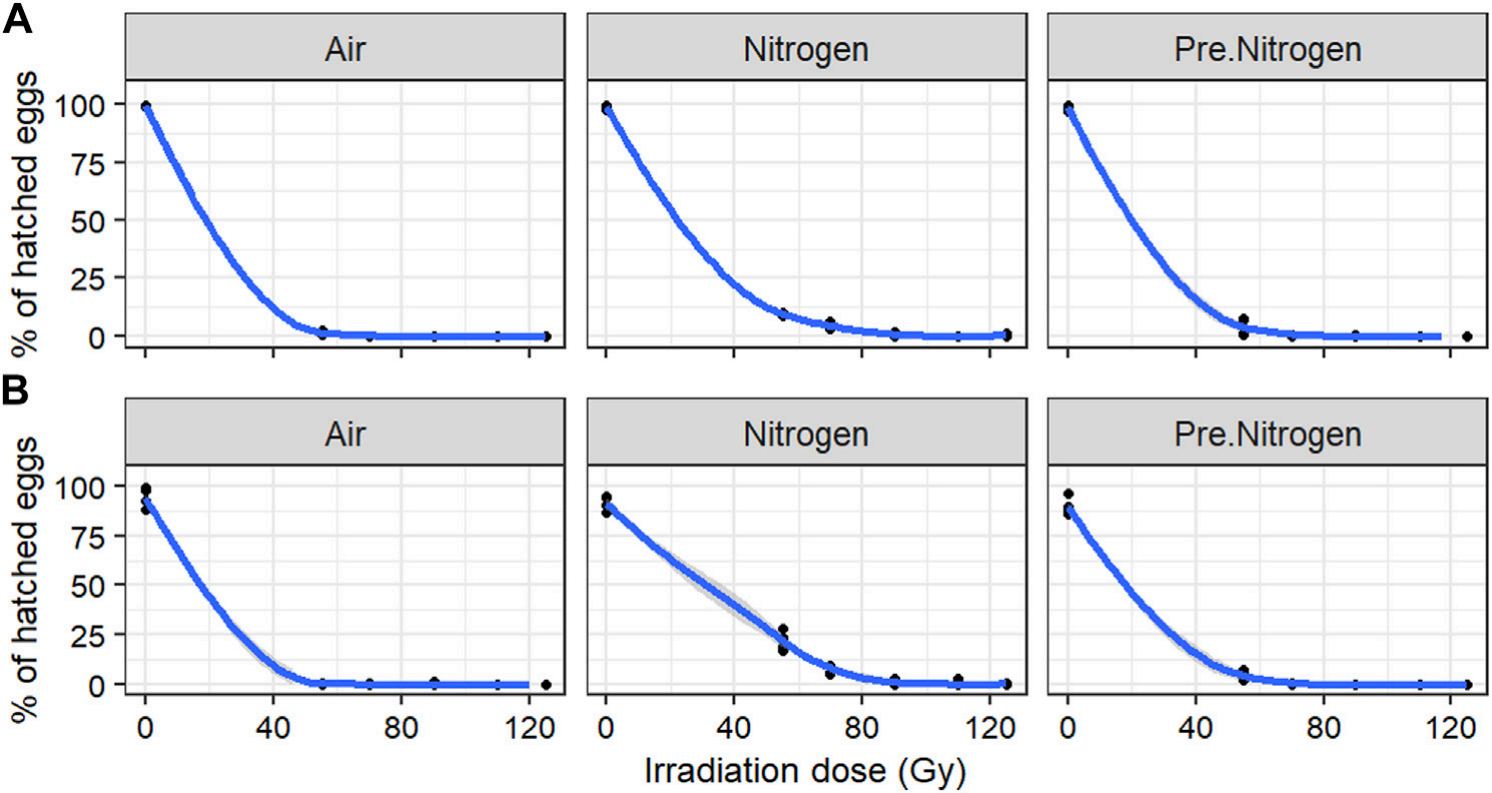
Egg hatch rate in *Aedes aegypti* **(A)** and *Aedes albopictus* **(B)** following irradiation of adult males at low density in different nitrogen environments.

The dose–response curves of *Ae. aegypti* and *Ae. albopictus* are presented in Figures 3A and B, respectively. Overall, the irradiation dose and environment had significant effects on the egg hatch rate (Table 2). In the absence of irradiation, the egg hatch rates in the three environments (air, PreN_2,_ N_2_) were above 98% in *Ae. aegypti* and above 85% in *Ae. albopictus.* The PreN_2_ and N_2_ treatments significantly decreased the radiosensitivity, with a higher effect observed with N_2_. An approximate of 0% egg hatch rate was reached with doses above 55 Gy in air, 70 Gy in PreN_2_, and 90 Gy in N_2_ in both species. However, full sterility was observed in all irradiation environments with doses of 110 and 120 Gy.

**TABLE 2:** Fixed-effects coefficients of binomial generalized linear mixed-effect models of the impact of irradiation dose and environment on the egg hatch rate in *Aedes aegypti* and *Aedes albopictus* following irradiation of adult males at low density.

Species		Estimate	Std. Error	z value	Pr (>|z|)
*Aedes aegypti*	(Intercept)	20.4104	1.6928	12.057	<2e-16 ***
	log (Dose)	−5.9556	0.4124	−14.441	<2e-16 ***
	Air	−1.2703	0.2875	−4.418	9.94e-06 ***
	Nitrogen	1.3672	0.1615	8.468	<2e-16 ***
*Aedes albopictus*	(Intercept)	20.3319	1.2006	16.93	<2e-16 ***
	log (Dose)	−5.9468	0.2892	−20.57	<2e-16 ***
	Air	−1.1588	0.2971	−3.9	9.6e-05 ***
	Nitrogen	2.2959	0.1723	13.33	<2e-16 ***

The treatment Pre.Nitrogen was set as the reference level (relevel) in the statistical analysis model.

Significant differences between treatment groups and the relevel group are indicated (*p < 0.005, **p < 0.01; ***p < 0.001).

### 3.3 Effect of irradiation of adult male *Aede*s at high density in different nitrogen environments on egg hatch rate and male survival

#### 3.3.1 Effect on egg hatch rate

**FIGURE 4:**
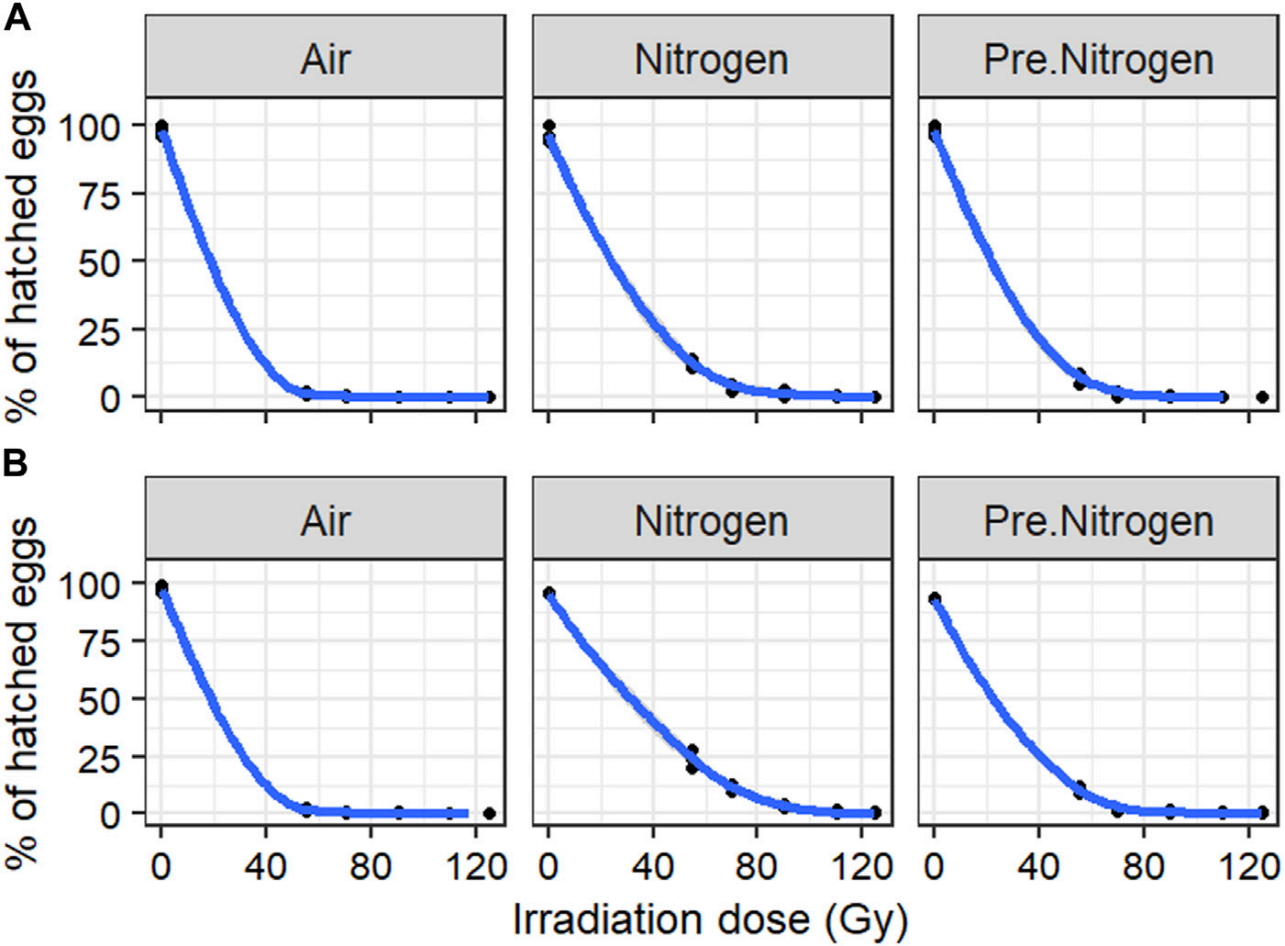
Egg hatch rate in *Aedes aegypti* **(A)** and *Aedes albopictus* **(B)** following irradiation of adult males at high density in different nitrogen environments.

Figures 4A and B present the dose–response curves from high-density irradiation for *Ae. aegypti* and *Ae. albopictus*, respectively*.* The egg hatch rates showed the same trend as that observed in the low-density irradiation in each species, although a slight increase was recorded in the PreN_2_ and N_2_ treatments at doses of 55, 70, and 90 Gy (Figure 4; Table 3). In both species*,* approximately 0% egg hatch rate was reached with doses above 55 Gy in air, 70 Gy in PreN_2_, and 90 Gy in N_2_.

**TABLE 3:** Fixed-effects coefficients of binomial generalized linear mixed-effect models of the impact of irradiation dose and environment on egg hatch rate in *Aedes aegypti* and *Aedes albopictus* following irradiation of adult males at high density.

Species		Estimate	Std. Error	z value	Pr (>|z|)
*Aedes aegypti*	(Intercept)	20.7311	2.7279	7.6	2.97e-14 ***
	log (Dose)	−5.8311	0.6645	−8.776	<2e-16 ***
	Air	−1.7315	0.4911	−3.526	0.000422 ***
	Nitrogen	0.7707	0.2439	3.16	0.001577 **
*Aedes albopictus*	(Intercept)	16.9846	1.6355	10.385	<2e-16 ***
	log (Dose)	−4.7993	0.394	−12.182	<2e-16 ***
	Air	−1.6241	0.3714	−4.373	1.22e-05 ***
	Nitrogen	1.1796	0.188	6.273	3.54e-10 ***

Pre.Nitrogen was set as relevel in the irradiation environment.

Significant differences between treatment groups and the relevel group are indicated (*p < 0.005, **p < 0.01; ***p < 0.001).

#### 3.3.2 Effect on male flight ability

**FIGURE 5:**
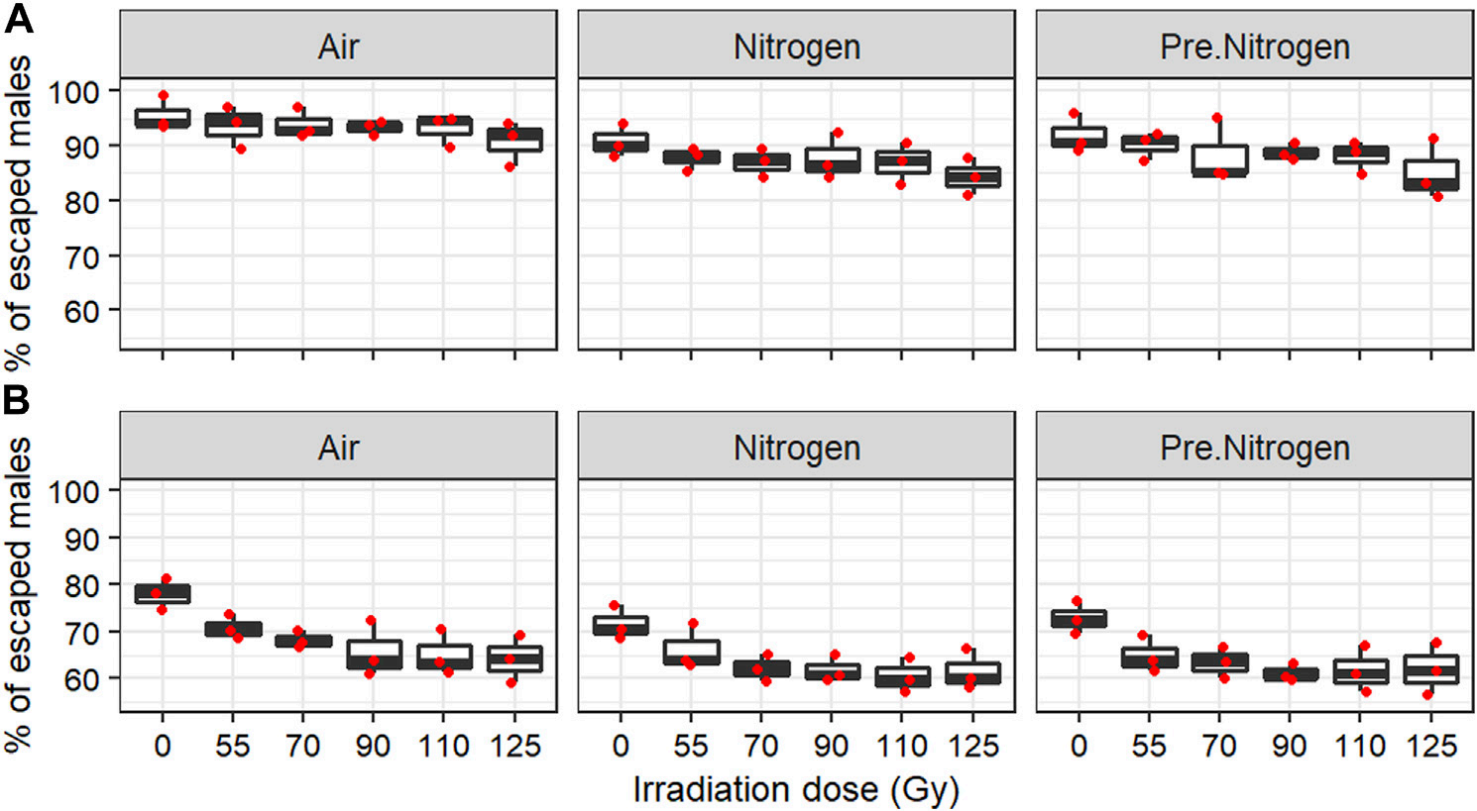
Flight ability of *Aedes aegypti* **(A)** and *Aedes albopictus* **(B)** following irradiation of adult males at high density in different nitrogen environments. Boxplots present the median values and quartiles, horizontal bars the 95% percentiles, black dots the minimal and maximal values, and red dots the replicate values.

Overall, the male flight ability (percentage of escapees) ranged between 80% and 100% for *Ae. aegypti* (Figure 5A) and between 55% and 85% for *Ae. albopictus* (Figure 5B). In both species, irradiation significantly reduced the male flight ability independently from the irradiation environment. In addition, irradiation in air showed better male flight ability than the PreN_2_ and N_2_ treatments regardless of the dose. The two nitrogen treatments had similar effects (Table 4). However, the relative differences observed in the male flight ability between the doses inducing nearly full sterility in air (55%) and in PreN_2_ (70%) and N_2_ (90%) treatments were only approximately 10%.

**TABLE 4:** Fixed-effects coefficients of binomial generalized linear mixed-effect models of the impact of irradiation dose and environment on male flight ability in *Aedes aegypti* and *Aedes albopictus* following irradiation of adult males at high density.

Species		Estimate	Std. Error	z value	Pr (>|z|)
*Aedes aegypti*	(Intercept)	2.48643	0.15681	15.856	<2e-16***
	log (Dose + 1)	−0.10564	0.02425	−4.357	1.32e-05 ***
	Air	0.53563	0.09424	5.683	1.32e-08***
	Nitrogen	−0.12095	0.08141	−1.486	0.137
*Aedes albopictus*	(Intercept)	1.00673	0.08317	12.105	<2e-16***
	log (Dose + 1)	−0.11073	0.01772	−6.248	4.16e-10 ***
	Air	0.19682	0.06873	2.864	0.00419**
	Nitrogen	−0.01529	0.06813	−0.224	0.82241

Pre.Nitrogen was set as relevel in the irradiation environment.

Significant differences between treatment groups and the relevel group are indicated (*p < 0.005, **p < 0.01; ***p < 0.001).

#### 3.3.3 Effect on male survival

**FIGURE 6:**
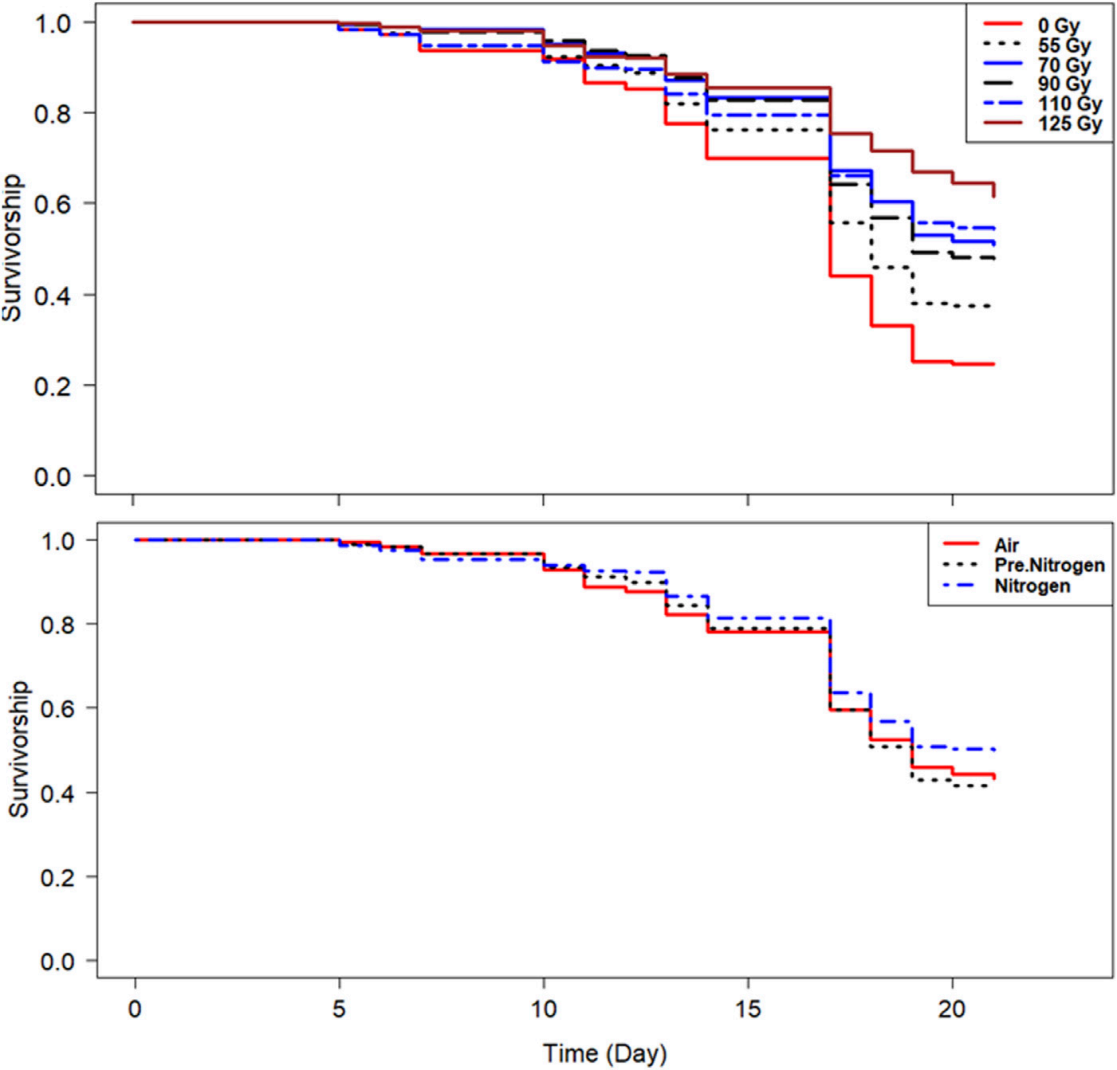
Survival of *Aedes aegypti* following irradiation of adult males at high density in different nitrogen environments.

**FIGURE 7:**
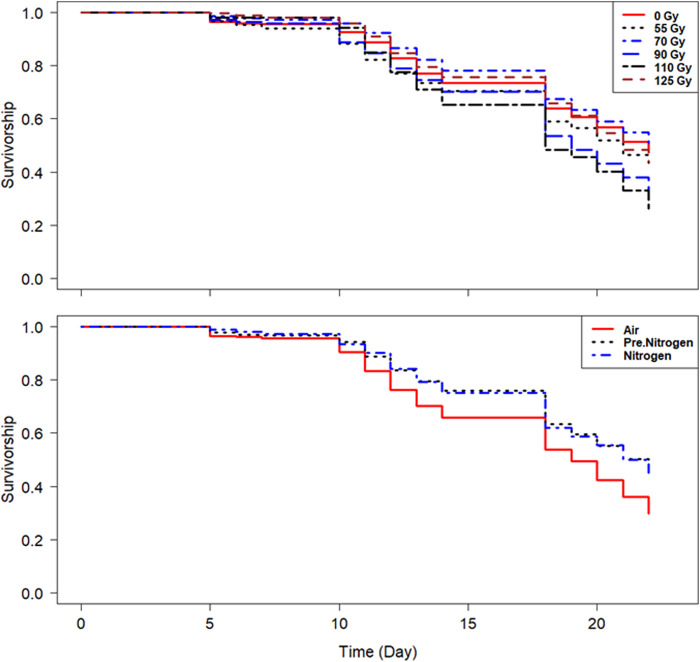
Survival of *Aedes albopictus* following irradiation of adult males at high density in different nitrogen environments.

In both species, the survivorship was above 90% on day 10 and above 70% on day 15 in all treatments (Figures 6, 7). In addition, the PreN_2_ and N_2_ treatments had similar positive effects on survival compared with irradiation in air (Figures 6, 7; Table 5), except for the PreN_2_ treatment in *Ae. aegypti*, which did not have a significant impact on longevity.

**TABLE 5:** Fixed-effects coefficients of mixed-effects Cox regression models of the impact of irradiation dose and environment on male longevity in *Aedes aegypti* and *Aedes albopictus* following irradiation of adult males at high density.

Species		coef	exp (coef)	se (coef)	z	*p*
*Aedes aegypti*	Dose	−0.0079192	0.992112	0.000616	−12.86	<0.0001
	Air	0.1398707	1.150125	0.064035	2.18	0.029
	Nitrogen	0.0416091	1.042487	0.064992	0.64	0.52
*Aedes albopictus*	Dose	0.0029937	1.002998	0.000625	4.79	1.60E-06
	Air	0.3900715	1.477086	0.05996	6.51	7.70E-11
	Nitrogen	0.010709	1.010767	0.063345	0.17	8.70E-01

Pre.Nitrogen was set as relevel in the irradiation environment.

Significant differences between treatment groups and the relevel group are indicated (*p < 0.005, **p < 0.01; ***p < 0.001).

## 4 Discussion

Ionizing radiation is the method of choice for the sterilization of male mosquitoes in SIT programs (Helinski et al., 2009). Radiation-induced sterility is the result of dominant lethal mutations in the germ cells caused by radiation (LaChance, 1967). Although germ cells are more sensitive to radiation and are targets for mosquito sterilization, damage also occurs in somatic cells, especially in those undergoing mitotic division, leading to reduced quality traits such as longevity and competitiveness (Proverbs, 1969). For the SIT to be successful, it is important to minimize the negative effects of irradiation. Irradiation in low-oxygen environments (for example, in nitrogen) has been reported to have a radioprotective effect with a beneficial impact on the quality of sterile males in many insect species (Vreysen, 1995; Fisher, 1996; Mutika and Parker, 2006). However, its effect on mosquitoes was not clear (Hallinan and Rai, 1973; Curtis, 1976; El-Gazzar et al., 1983; Yamada et al., 2022) and needed to be investigated further.

The present study investigated the possibility to use nitrogen for adult immobilization in the mass irradiation of *Ae. albopictus* and *Ae. aegypti* without inducing adverse effects on their quality. Overall, significant differences were observed between the irradiation environments in the dose–response curves of the egg hatch rate within each species. The doses needed to achieve acceptable sterility were, by order of increase, higher in PreN_2_ and N_2_ compared with that for irradiation in air. These results are consistent with the results of previous studies that reported that higher radiation doses were required under nitrogen treatments to achieve adequate induced sterility in many insects, including fruit flies, tsetse flies, and mosquitoes (Hallinan and Rai, 1973; Curtis, 1976; El-Gazzar et al., 1983). Oxygen is known to be a radiosensitizer (Forshier, 2012). Therefore, the high sensitivity of insects to ionizing radiation in air is commonly attributed to the high level of oxygen. In contrast, the radioresistance under N_2_ is likely because of the absence or low level of oxygen in the cells, as reported previously (Condon et al., 2017; Sassù et al., 2019). The radioresistance observed in males pretreated with nitrogen is also likely because of low oxygen saturation in the tissues during irradiation, which is also the main mechanism responsible for paralysis, which can last for ∼15 min after removing the N_2_, and a lower metabolic rate. Immobilization of mosquitoes in nitrogen decreases the physiological and biochemical reactions that normally interact with radiation, resulting in radioresistance, since cells that are actively undergoing mitosis are known to be more sensitive to radiation (Proverbs, 1969). The hypothesis of anesthesia-induced radioresistance is supported by studies that assessed the effect of temperature during irradiation. Indeed, it has been reported that lower temperatures decreased the induced sterility of *Ae. aegypti* irradiated at the pupal or adult stages (Ernawan et al., 2022).

In our study, irradiation of high-density adult male *Aedes* either in air or in nitrogen treatments had similar extents of induced sterility as irradiation at low mosquito density. This result shows the potential of using nitrogen in adult mass irradiation. However, besides induced sterility, good quality of males is an important requirement to achieve the goal of the SIT.

Our results showed that the male flight ability was reduced by irradiation and that the effect was more pronounced under nitrogen treatments. These results corroborate those of previous studies that reported a negative effect of high ionizing radiation on sterile male quality, commonly attributed to the deleterious effect provoked in somatic cells (Forshier, 2012; Culbert et al., 2018). A recent study also found a reduction in the male flight ability of *Ae. albopictus* irradiated at 45 Gy in nitrogen compared with irradiation in normoxia as well as unirradiated males (Yamada et al., 2022). However, in our study, the relative differences observed in the male flight ability between the doses inducing nearly full sterility in air and nitrogen treatments were only approximately 10%. Therefore, it remains to be elucidated whether this extent of difference would affect the relative male competitiveness. Indeed, adult male *Ae. aegypti* irradiated at 100 Gy in nitrogen were found to be fully sterile and were as competitive as unirradiated males, while males irradiated in air at 35, 70, or 100 Gy were less competitive (Hallinan and Rai, 1973). A longer recovery time may enhance the flight ability of the males irradiated under nitrogen treatments and improve their competitiveness, as the flight tests were performed only 1 day after irradiation in the current study. This hypothesis is supported by the beneficial effect of irradiation in nitrogen treatments observed on male survival, as expected (Vreysen, 1995; Fisher, 1996). Overall, high survivorship (>90%) was recorded at day 10 postemergence (7–8 days after irradiation) regardless of the irradiation dose and environment. This suggests that the lifespan of males irradiated in our study conditions would be suitable for the SIT programs as male mosquitoes exhibit a higher mating ability within 10 days after emergence (Sawadogo et al., 2013; Damiens et al., 2016).

Furthermore, this study showed that PreN_2_ treatment could be a reliable method for immobilizing adult mosquitoes for irradiation. Indeed, in contrast to the control, where mobility was observed during the irradiation period, mosquitoes stayed immobilized in both types of nitrogen treatment. However, long-term immobilization could be detrimental to mosquitoes. The best option would thus be the PreN_2_ treatment as it reduces the immobilization duration, and it requires a lower dose than that required in N_2_ environments to achieve full sterility but with similar effects on male quality.

Nitrogen treatment requires additional methods such as a cooling system to knock down the mosquitoes for compaction before the nitrogen treatment as well as for handling and transport purposes after irradiation. It, however, reduces the duration of chilling and allows conduction of the irradiation process without reducing the temperature in the irradiator chamber. For the optimal use of nitrogen, further studies are necessary to develop standardized procedures including the type of container, time and pressure for filling nitrogen, and immobilization duration, considering mosquito species, age, and density.

## Data Availability

The raw data supporting the conclusion of this article will be made available by the authors, without undue reservation.
